# Noninvasive Diagnosis of Chemotherapy Related Cardiotoxicity

**DOI:** 10.2174/157340311799960672

**Published:** 2011-11

**Authors:** Hamilton S Gillespie, Christopher J McGann, Brent D Wilson

**Affiliations:** * Cardiovascular Center, University of Utah, Salt Lake City, Utah 84132

**Keywords:** Noninvasive imaging, nonischemic cardiomyopathy, chemotherapy-related cardiotoxicity.

## Abstract

Chemotherapeutic agents reduce mortality and can prevent morbidity in a wide range of malignancies. These agents are, however, associated with toxicities of their own, and the treating physician must remain ever vigilant against the risk outgrowing the benefit of therapy. Thus, pre-treatment evaluation and monitoring for toxicity during and following administration is a fundamental tenet of oncologic practice. Among the most insidious and deadly toxicities of anti-tumor agents is cardiac toxicity, which in some cases may be irreversible. Early detection of cardiotoxicity allows the treating oncologist to redirect therapy or dose modify, taking into account the cost of a reduction in therapy against the potential of further injury to the patient. In these instances, the role of the cardiologist is to assist and advise the oncologist by providing diagnostic and prognostic information regarding developing cardiotoxicity. This review discusses noninvasive diagnostic options to identify and characterize cardiotoxicity and their use in prognosis and guiding therapy. We also review established protocols for cardiac safety monitoring in the treatment of malignancy.

## INTRODUCTION

Over the past decade a bonanza of new chemotherapeutic agents have matured, in many cases rendering cancer a chronic disease rather than a terminal one. As early detection of malignancy leads to a higher likelihood of cure, the treated population may survive to experience secondary toxicities of their cancer therapy, in spite of the initial benefit. Of paramount importance in both morbidity and mortality are the short and long term effects of cancer therapy on cardiac tissue and subsequent effects on cardiac function. Early detection of toxicity during therapy may allow for dose modification, alteration, or abandonment of regimens which may reduce or even eliminate long term complications associated with reduced left ventricular function. Further, there is mounting evidence that early therapeutic interventions with cardioprotective medications such as beta-blockers or angiotensin-renin-aldosterone system altering agents may improve cardiovascular outcomes in the population treated with chemotherapeutic agents [1, 2]. Non-invasive evaluation of the cardiovascular system prior to initiation of therapy may also provide prognostic information and influence the decision making of the oncologist. The role of the consulting cardiologist is therefore to provide diagnostic information to the oncologist in support of the best possible outcome for the patient both in relation to their malignancy and to their long term cardiovascular morbidity and mortality, as well as to support patient monitoring during and after therapy in order to identify cardiovascular complications early.

It is important to note that the demographics and exposure history of many oncologic patients is such that the incidence of cardiovascular disease is higher than in other populations. Oncologic patients frequently have one or more risk factors for coronary artery disease (age > 55 for men, age > 65 for women, smoking history, hyperlipidemia, hypertension, CAD family history). Regarding clinical monitoring for cardiotoxicity in clinical trials of chemotherapeutic agents, the Cardiac Review and Evaluation Committee (CREC) reviewing cardiotoxicity in several trials of trastuzumab in the treatment of breast cancer formulated the following, which has become the de facto standard: cardiomyopathy is characterized by [1] a decrease in cardiac left ventricular ejection fraction (LVEF) that was either global or more severe in the septum; [2] symptoms of congestive heart failure (CHF); [3] associated signs of CHF, including but not limited to an S3 gallop, tachycardia, or both; [4] a symptomatic decline in LVEF of at least 5% to an LVEF of less than 55%, or an asymptomatic decline in LVEF of at least 10% to an LVEF of less than 55% [3]. Though this model does provide a reasonable standard, it is by no means universal. Further, the toxic effects of chemotherapeutic agents on the heart may be more varied than a simple reduction in left ventricular systolic function, such as induction of cardiac ischemia, valvular, or electrical dysfunction. These potential complications are not captured by a model of toxicity monitoring focused only on a reduction in LV function.

The nature of the chemotherapeutic agent plays a central role in determining cardiotoxicity. Cardiotoxicity may occur *via* several mechanisms, including a) direct cellular toxicity involving cardiac myocytes, resulting in both systolic and diastolic dysfunction; b) induction of ischemia through vasoactive side effects, thrombogenesis, or vascular toxicities; c) arrhythmia either as a collateral effect to cellular toxicity or due to interference with cellular membrane channels; d) induction of myocardial and/or pericardial inflammation (myopericarditis) with associated direct dysfunction or mechanical sequelae due to a pericardial effusion. Survival can be markedly improved with intervention, both in the form of dose adjustment if cardiotoxicity is recognized early, or with medical therapy to allay the onset of heart failure and perhaps retard its progression. With many of the agents described below, when myocardial dysfunction is present in the chronic phase, it commonly represents permanent myocyte injury, and though the syndrome may be clinically treatable for a time the pathophysiology is most likely irreversible. This belies the importance of early detection of toxicity, in the hopes of preventing disease progression. 

## INITIAL ASSESSMENT

Assessment of potential toxicity begins with physical examination and historical features, including an assessment of functional status. A thorough history and physical examination is paramount. Clinical predictors including age, prior hypertensive, vascular, or other cardiac disease are powerful predictors of subsequent toxicity. Other readily available clinical parameters such as biochemical markers, electrocardiographic studies, and finally noninvasive imaging, provide further diagnostic power in determining the presence, degree, and progression of cardiac toxicity. Intensive monitoring of cardiotoxicity in chemotherapeutic regimens may allow for early detection of cardiac dysfunction, and thereby allow clinical intervention, either by dose adjustment, pharmacologic intervention, regimen modification, or therapy aimed at treating subsequent heart failure as it develops. Further, regimens or agents administered prior to the infusion of anthracycline may induce myocardial dysfunction, which makes the patient more susceptible to a clinically significant decline in cardiac function. The protocol under which the anthracycline was infused also has an impact on the likelihood of subsequent development of cardiotoxicity; bolus protocols tend toward cardiotoxicity more than slow infusion protocols, perhaps unveiling a toxic threshold within the mechanism of anthracycline-induced cardiotoxicity [4, 5] The anthracycline agent itself also influences the degree of cardiotoxicity, with doxorubicin being the most toxic, and epirubicin and mitoxantrone less so. Similarly, modification of the delivery vehicle of the chemotherapeutic agent may reduce cardiotoxicity later on -- such as with liposomal encapsulation of the anthracycline molecule -- as may administration of protective agents such as dexrazoxane, discussed elsewhere. The clinical history must therefore assess and outline these features, in understanding the likely risk of chemotherapy-associated cardiotoxicity. 

## CHEMOTHERAPEUTIC AGENTS

The nature of the chemotherapeutic agent in use is an important factor in determining the likelihood and natural history of cardiotoxicity, if it develops. Comprehensive discussion of the specific cardiotoxicities of the various chemotherapeutic agents are discussed elsewhere in this series. In summary, the mechanism of cardiotoxicity may result from direct injury to the cardiomyocyte, ischemia either due to native disease or vascular effects of the agents in question [2, 6-8]. These agents include the anthracyclines such as doxorubacin, antimetabolites such as 5-fluorouracil and cytarabine, alkylating agents such as cyclophosphamide, and microtubule agents such as vincristine, anti-tumor antibiotics or other biologicals such as trastuzumab. In each case, cardiomyocyte death with subsequent fibrosis within the myocardium produces myocardial dysfunction, which is the most well-described and dreaded long term complication of toxicity. In its various modalities, noninvasive cardiovascular imaging provides a window into the chest, with visualization of cardiac structure, wall motion, myocardial perfusion, and myocardial histology. An understanding of the pathologic mechanisms is an important component of assessment and in deriving a noninvasive monitoring strategy.

## ENDOMYOCARDIAL BIOPSY

Though not a noninvasive procedure, endomycardial biopsy does deserve some mention here. In patients with cardiac toxicity, electron microscopy of endomyocardial biopsy specimens reveal disruption of z-lines, mitochondrial disruption, myofibrillary bundles, myofibrillar lysis and vacuolization within the myocytes [43]. Though the endomyocardial biopsy is generally regarded as the de facto standard in the diagnosis of non-ischemic heart failure, obtaining myocardial tissue for biopsy requires an invasive procedure. Endomyocardial biopsy is generally restricted to cases in which a high degree of certainty is sought and requires evaluation by pathologists experienced in interpreting appropriate findings. The procedure consists of passing a small bioptome repeatedly *via* a central vein (commonly the right internal jugular) across the tricuspid valve and into the right ventricle. There, several small biopsies are taken from various positions on the interventricular septum. Though uncommon, complications can occur and include perforation of the right ventricular free wall with subsequent hemopericardium and possible development of tamponade physiology requiring emergent drainage. Other complications include arrhythmia, injury to the tricuspid valve, fistula formation, bleeding and infection. The nature of cardiotoxicity in the setting of many chemotherapeutic agents often occurs in a patchy fashion, thus multiple (3-5) biopsies are required, and even in this setting the sensitivity of endomyocardial biopsy in diagnosing chemotherapy-induced cardiotoxicity is low. Specificity is, however, quite high and can confirm the diagnosis. 

## BIOMARKERS

Cardiac biomarkers generally refer to proteins released from cardiac myocytes under conditions of both mechanical and severe metabolic stress, up to and including cell death. Myocyte death, or severe metabolic stress, will lead to the release into the blood of intracellular proteins, including AST, ALT, creatine-kinase (CK) and the cardiac isoenzyme of CK (CK-MB), as well as cardiac isoforms of troponins I and T (cTnI and cTnT). While skeletal muscle may encode at least trace amounts of cardiac CK-MB, the exclusive translation of the cardiac troponins within the cardiac myocyte renders them more accurate for the detection of cardiac damage. Cardiac troponin T and I are released from cardiac myocytes under conditions of severe metabolic stress, but more commonly following cell death and eventual lysis. These markers reach the bloodstream where they may then be measured peripherally. Cardiac troponins are established as a sensitive and specific marker of cardiac injury. B-type naturetic peptide is a sensitive biochemical marker of myocardial stretch and strain, and has been validated as a diagnostic tool in identifying congestive heart failure as the source of dyspnea [9]. Mechanical strain within the atria and ventricles leads to release of atrial natriuretic peptide and B-type natriuretic peptide, respectively. These peptides function as hormones and interact with nephrons, dominantly within the distal tubule to affect diuresis and to regulate the intravascular volume state. 

Elevations of cardiac biomarkers have been closely associated with acute toxicities attributable to chemotherapeutic agents, however evidence is lacking to support long term associations of toxicity as well as the prospective use of cardiac biomarkers in determining a course of therapy. Nonetheless, cardiac biomarkers may play a central role in the early identification of patients at high risk for cardiac toxicity in the setting of chemotherapy [10]. Though other biomarkers may be useful in various contexts, the bulk of evidence in predicting and following cardiotoxicity related to chemotherapeutic agents lies with measurements of cardiac troponin and BNP. A major advantage of the use of cardiac biomarkers lie in their reduced cost, convenience, reproducibility, and lack of radiation or contrast exposure when compared to many of the imaging modalities. 

**Fig. (1):**
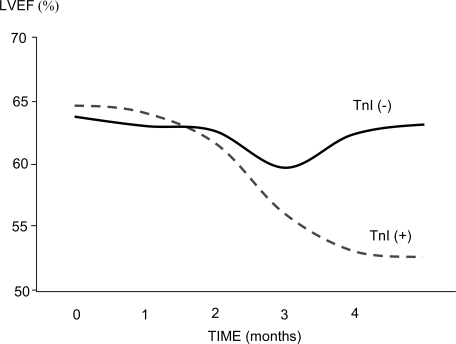
Relationship of left-ventricular ejection fraction, as assessed by echocardiography, in patients undergoing high-dose chemotherapy. Those with any elevation in troponin-I above a cutoff of 0.5 ng/mL after any cycle of chemotherapy demonstrated a significant and persistent drop in left ventricular ejection fraction after roughly 3 months, an effect which persisted out to 7 months. (*adapted with permission from Cardinale D et al. JACC 2000; 36(2):517-522*)

During the administration of high-dose chemotherapeutic regimens, elevations in cardiac troponins correlate strongly with the development of cardiac dysfunction in patients treated with high dose chemotherapy (see Figure 1) [11,12]. In animal models of anthracycline-induced cardiotoxicity, serum troponin T concentration correlated with cumulative dose of drug received as well as assessed myocardial damage [13]. Pediatric series have further confirmed this correlation [14]. Both acute and persistent rises in cardiac troponins following chemotherapy out to one month have been associated with a higher incidence of left ventricular dysfunction and adverse cardiovascular events within one year, as well as long term reductions in left ventricular function beyond 40 months [10, 11, 15]. The negative predictive value of the troponin assay shows promise, however larger and more diverse studies are required to rely upon this biomarker as a means of excluding patients from long-term surveillance. Further, the timing to peak troponin value is not clearly defined, and serial measurement immediately following chemotherapy administration, and at 12, 24, 36, and 72 hours, may be required to detect these elevations [12]. This may limit the clinical utility of troponin assays to those patients who are either hospitalized or receiving daily follow-up immediately after their chemotherapy cycle. 

In the case of BNP assays - both direct measurement of serum BNP as well as NT-pro-BNP - persistently elevated serum BNP measurements correlate with noninvasive measurement of left ventricular dysfunction. NT-proBNP has been shown to consistently rise above threshold levels with administration of 5-FU, and to rise more so in those patients who experience anginal symptoms associated with their 5-FU infusion [16]. As with heart failure in the general population, however, an accurate cutoff has been elusive. Measurement of post-treatment BNP levels may help in identifying patients at high risk of later manifestation of cardiotoxicity. Measures of either cardiac troponins or BNP cannot, however, supplant noninvasive imaging of left ventricular function.

## ELECTROCARDIOGRAPHY

The advantages of 12-lead electrocardiography (ECG) are that the procedure is inexpensive, widely available, and without risk to the patient. ECG is essential in the diagnosis of heart rhythm abnormalities. In addition, reduction in limb lead voltages by >30% may predict cardiotoxicity in patients receiving anthracyclines [44]. However, non-invasive assessment of left ventricular function is widely accepted as a more sensitive means of detecting early cardiac toxicity. 

## CARDIOVASCULAR IMAGING

Evaluation and surveillance of cardiac function is recommended before, during, and after administration of chemotherapeutic agents with associated cardiotoxicity. There are not, however, published consensus guidelines on the modality or frequency of cardiac assessment - nor on the most effective parameter or set of parameters to follow in a particular situation. Further, coronary artery disease is prominent among many populations of cancer patients, and non-invasive cardiovascular imaging may be used to assess for significant coronary artery disease in addition to assessing for toxicity. Comparison between imaging of both cardiac structure and of perfusion or perfusion surrogates during both rest and stress conditions is a widely utilized paradigm for assessment of injured or ischemic myocardium.

The most common parameter assessed *via* noninvasive imaging in this context is the left-ventricular ejection fraction (LVEF). Often interpreted as an aggregate of left ventricular systolic function, the ejection fraction represents the fraction of left ventricular blood volume at end diastole that is ejected during systole (often expressed as a percentage). Its assessment relies upon accurate evaluation of the left ventricular chamber volume at these two points in the cardiac cycle. Early data, particularly in patients treated with anthracyclines, demonstrated a fall in LVEF associated with the development of anthracycline associated persistent cardiomyopathy. A minor (≤4%) fall in LVEF early in the course of anthracycline based chemotherapy, below what is considered the standard cumulative toxic dose in adults, has also been demonstrated to predict later sustained fall in EF into the toxic threshold in treated patients [17]. Thus, accurate, reproducible, and safe noninvasive assessment of LVEF becomes an important component of monitoring patients undergoing chemotherapy. However, a significant amount of myocardium must be involved to produce a consistently measurable fall in overall systolic function and to be reflected in the aggregate ejection fraction. 

As cardiovascular disease, dominantly ischemic heart disease, remains the most common cause of death in the western world, much of what is known about the non-invasive imaging of cardiac structure, function, and dysfunction is derived from efforts to understand and noninvasively detect myocardial ischemia. As the decoupling of myosin from actin is an energy dependent process, the initial myocyte dysfunction in the ischemic heart induces a decline in diastolic function. This leads to an elevation in filling pressures and activation of stretch receptors within the ventricular (and atrial) walls, subsequently activating the neuroendocrine axis and leading to a release of natriuretic peptides. Loss of systolic function may occur as ischemia progresses to infarction and myocardial necrosis; autopsy studies have in fact demonstrated patchy areas of myocyte necrosis in such patients. Assessment of other parameters of myocardial motion throughout the cardiac cycle, as well as visualization of myocardial tissue features, may provide clues to subclinical toxicity well before it is reflected by a fall in ejection fraction. The strengths and weaknesses of several methods of assessing left ventricular function are therefore described below. 

## NUCLEAR MEDICINE STUDIES

Early studies of doxorubicin cardiotoxicity relied upon radionucleotide ventriculography to risk stratify patients pre-therapy, and to follow patients serially during therapy in hopes of identifying early signs of cardiotoxicity. 

Radionucleotide ventriculography (RVG) is often performed *via* equilibrium gated techniques to provide an accurate estimate of left ventricular volumes at end-diastole and end-systole, thus providing a surrogate metric of left ventricular function. A radiolabel (commonly technetium 99m) is attached to red blood cells or serum albumin from a patient blood sample and re-injected. In the multiple-gated acquisition technique (MUGA), the summation of counts from 800-1000 ECG-gated cardiac cycles is performed, producing an average cardiac cycle with good resolution of LV chamber dimensions. These dimensions are acquired in 3 standard projections - anterior, left anterior oblique, and left lateral. Each projection requires roughly 5 minutes of imaging time to acquire. Attempts to compare radionucleotide ventriculography (RVG) to standard 2D and M-mode echocardiography techniques have demonstrated inexact correlation [18]. Serial measurements of LVEF by RVG have been shown to be highly reproducible. However, RVG requires a significant radiation dose (estimated at 7.8 mSv per examination) with each assessment, and is generally more costly than echocardiography [19]. Unlike echocardiography where there is direct visualization of the myocardium and cardiac structures, RVG provides little to no information about valvular function and other cardiac parameters. Thus RVG is presently used less often than echocardiography in assessment and monitoring for chemotherapy-induced cardiotoxicity. It is a reasonable option when poor echocardiographic windows preclude assessment with ultrasound, or in confirming a decline in left ventricular function identified with echocardiography. Further, RVG may have a role to increase accuracy of LVEF assessment in patients who are at higher risk for development of cardiac toxicity, such as individuals treated with higher doses of anthracyclines, or significant doses of mediastinal radiation (greater than 10 Sieverts) for lymphoma or leukemia in childhood [20].

Other nuclear medicine modalities may be used to evaluate myocardial perfusion and estimate ischemic burden due to coronary artery disease. These include ^99m^Technetium-labelled tracers as well as ^201^Thallium chloride. This group of myocardial perfusion studies rely on differences in regional myocardial blood flow to identify hemodynamically significant coronary stenosis, as well as uptake of the tracer by metabolically active tissue to assess myocardial viability. Radiation dose estimates with these modalities range from 9.4-12.8 mSv for a ^99m^Technetium-sestamibi myocardial perfusion scan to as high as 40.1 mSv for a ^201^Thallium viability study [21].

To put radiation exposure in context, medical workers and individuals in other occupations which expect repeated exposure to low-dose ionizing radiation typically limit radiation doses to 100 mSv every five years, with a limit of no more than 50 mSv in any given year period. The estimated average annual exposure to ionizing radiation worldwide is 2.4 mSv. Thus, a single study carries perhaps less concern in regard to long-term risk of ill effect, however repeated exposures, for example as part of a surveillance protocol, may be more concerning. The BEIR VII Phase 2 committee estimated an increased lifetime risk of 800 solid malignancies and 100 leukemias per 100,000 persons would result from a single exposure to 100 mSv [22]. The risk estimate due to repeated lower level exposures is as of yet unknown. 

## ECHOCARDIOGRAPHY

Echocardiography is the most commonly utilized non-invasive modality for assessing cardiac structure and function. The advantages of echocardiography over other methods of cardiovascular imaging include availability, lesser cost, lack of ionizing radiation and lack of exposure to potentially toxic contrast agents. In addition to accurate assessment of left ventricular function, echocardiography provides information about valvular function, presence of pericardial fluid, pulmonary arterial pressures, left ventricular filling pressures, and intravascular volume status. These additional parameters, while they may or may not be related to chemotherapeutic administration, provide useful clinical information. Like other modalities, body habitus and anatomic variation (such as rib location) affect image quality, and there can be inter-observer differences in study interpretation. Newer techniques, such as 3D echocardiography and tissue Doppler velocity assessments are becoming more widely used and will improve the accuracy and reproducibility of cardiac ultrasound. For example, with 3D echocardiography, volume assessment can be performed without relying on geometric assumptions (which is the case with standard 2D echocardiography) and provides an accurate, quantitative means of assessing left ventricular function. Dobutamine or treadmill stress echocardiography may also have a role in identifying patients with cardiomyopathy, as these studies allow assessment of contractile reserve and response to increased workloads. 

Standard methods of 2D echocardiography can be used to assess left ventricular function. M-mode echocardiography refers to an evaluation of structures along a single dimensional line, followed over time. This mode allows for tremendous temporal resolution, thus allowing for evaluation of motion of various cardiac structures - in particular the left ventricular endocardium. By convention, the LV cavity dimensions are measured at the level of the mitral leaflet tips or papillary muscle using 2D-guided M-mode. From these measurements, the fractional shortening can be calculated as well as an estimate of the fraction of blood volume ejected with each cardiac cycle, the ejection fraction. Using 2D echocardiography, the endocardial border can be traced by the interpreting clinician in two orthogonal planes (usually from an apical 4-chamber and apical 2-chamber view). From these traced areas, the LV cavity volume may be estimated *via* the modified Simpson method (also termed the disk summation method). This method improves accuracy over M-mode estimates, and reduces inter- and intra-observer variability. These method does suffers from error in estimation of cavity size due to geometric assumptions which may not hold up from patient to patient, under different loading conditions, or when myocardial wall motion abnormalities have developed. Tissue velocity Doppler imaging (DTI) relies upon high-amplitude, low frequency Doppler shifts to measure velocity of various cardiac structures, including the left ventricular endocardium. Tissue velocities at different locations during various phases of the cardiac cycle are measured with this technique, and correlations to outcomes have been found in a number of disease states [23]. The velocity of the left ventricular endocardium (V_endo_) at various locations is observed and can be used as a surrogate for left ventricular function. In murine models of anthracycline-induced cardiotoxicity, V_endo_ allows earlier recognition of LV dysfunction when compared to standard 2D echocardiographic techniques (M-mode and 2D echo estimation of fractional shortening and ejection fraction) - as early as 1 day vs 4-5 days, respectively. Further, DTI produces lower rates of inter and intra-observer variability [24]. In patients undergoing therapy with anthracyclines for breast cancer, diastolic dysfunction (assessed by DTI) may serve as a more sensitive marker of toxicity. In a prospective study of patients receiving traztuzumab for breast carcinoma, evaluation of the tissue velocity of the lateral mitral annulus at 3 months predicted, with statistical significance, a fall in LVEF of greater than 10% at 6 months into therapy; peak longitudinal and radial strain *via* a speckle tracking technique were also significantly decreased early in this study [25]. DTI may improve the sensitivity of echocardiography for identifying diastolic dysfunction, and thus improve detection of chemotherapy-induced toxicity with anthracyclines [26]. 3D echocardiography improves estimation of LV volume and may provide improved accuracy, when compared to standard echocardiographic techniques [27]. With 3D echocardiography, endocardial borders are identified on multiple images and volumes are calculated. Techniques are available on newer echocardiography systems to better automate this process, thus reducing inter-observer and intra-observer variability. However, cumbersome data acquisition and significant offline processing requirements have limited the clinical utility of 3D echo to date. Negative bias, leading to an underestimation of LV cavity volumes both during systole and diastole, is reduced utilizing a 3D automated detection technique, and excellent correlation with volumes measured on cardiac MRI have been demonstrated. As faster computer processing power becomes more widely available, and transducer technology continues to advance, 3D echo will gain a larger presence in the clinical arena. 

Echocardiography is a safe imaging modality. The acoustic power of echocardiographic equipment is kept well below levels at which adverse effects of ultrasound energy may be observed. These effects include heating of target and intervening tissues, as well as induction of electrical dysfunction, often observed as frequent premature ventricular contractions (PVCs). Microbubble contrast agents can be administered in patients with difficult echo windows and make it possible to obtain diagnostic quality images in virtually all patients. Of the imaging techniques described here, echocardiography is the least expensive. Overall, echocardiography provides is an excellent method for serially evaluating left ventricular function and provides a significant amount of ancillary information, including valvular assessment, pericardial effusion detection, etc. There are no known long-term sequela from ultrasound evaluation, and the technology is relatively inexpensive and widely available.

## CARDIAC COMPUTED TOMOGRAPHY (CT)

Coronary artery disease (CAD) is pervasive in our society, and is an important consideration in the differential diagnosis of any patient with decreased left ventricular function. CAD is the leading cause of death in the United States for both men and women. Given the high prevalence of CAD, and the fact that patients receiving chemotherapy often have multiple CAD risk factors, it is frequently necessary to exclude ischemia as a cause of cardiomyopathy, even in situations where a temporal correlation exists between onset of the cardiomyopathy and administration of the potentially cardiotoxic agent. Stress imaging studies can help identify potential ischemia; however, interpretation of these studies can be complicated in the setting of decreased left ventricular function. New technology in CT scanning enables direct visualization of coronary arteries and unequivocal determination of presence or absence of coronary artery disease [28]. Coronary CT angiography (CCTA) provides information about soft and calcified plaque in the vessel lumen and in the vessel wall. This information regarding plaque composition and vessel wall often exceeds that provided by invasive cardiac catheterization without the risks of an invasive cardiac procedure (stroke, aortic trauma/dissection, coronary trauma/dissection). The *2010 Cardiac CT Appropriateness Criteria* advocate that CCTA is appropriate in any patient with low or intermediate CAD risk with CAD symptoms. It is also appropriate in patients without symptoms but with new-onset or newly diagnosed heart failure with low or intermediate CAD risk [29]. In addition to evaluation of epicardial coronary arteries, CCTA enables accurate assessment of left ventricular ejection fraction, cardiac chamber sizes, and other thoracic, non-cardiac structures. In patients with high risk of CAD, invasive coronary angiography is recommended, as this can be accompanied by percutaneous intervention (balloon angioplasty or stent placement). 

While CCTA enables sensitive detection of coronary artery disease, there are several practical considerations that limit its use in patients with cardiomyopathy. Protocols commonly employed with current generation multi-detector row CT scanners require low heart rates (50-60 bpm) in order to obtain diagnostic images. This frequently requires aggressive pre-study beta blockade. In patients with reduced LVEF and a compensatory tachycardia to maintain cardiac output, beta blockade can be a difficult proposition. Patients with cardiomyopathy and reduced LVEF frequently have co-exiting arrhythmias, such as atrial fibrillation or premature ventricular contractions. Such heart rate irregularity also limits the ability to obtain diagnostic CCTA images. CCTA requires administration of iodinated contrast, which comes with increased risk of contrast nephropathy in patients with decreased renal function. Another consideration is the need for ionizing radiation with coronary CT angiography (with typical exposures of 3-15 mSv). Strategies to reduce the radiation dose *via* spatial and temporal modulation of the amount of x-ray radiation used during the study have reduced the doses of ionizing radiation in coronary CT substantially [21]. Financial considerations can be another factor. Despite its demonstrated clinical utility, insurance payors have been slow to reimburse cardiac CT studies. 

## CARDIAC MAGNETIC RESONANCE IMAGING (CMR)

Cardiac magnetic resonance imaging (CMR) is considered the gold standard for noninvasive estimation of cardiac volumes and assessment of left ventricular function [30]. Multiple tomographic images are obtained during various temporal points in the cardiac cycle, and volume reconstruction is not dependent upon geometric models, as is the case with echocardiography and ventriculography. MRI can further reliably assess diastolic parameters with moderate accuracy when compared to two-dimensional Doppler echocardiography. A major advantage of cardiac MRI is its lack of exposure to ionizing radiation, as imaging is accomplished utilizing variations in magnetic fields and administration of small amounts of radiofrequency energy. Some patients with paramagnetic metal prostheses or with other devices such as cardiac pacemakers or internal cardiac defibrillators may not be candidates for MRI because of potential interaction with the strong MRI magnetic fields (1.5-3 Tesla). Patients with creatinine clearance <30 mL/min also may not be candidates for a cardiac MRI with gadolinium contrast because of the rare syndrome of nephrogenic systemic sclerosis. This scleroderma-like syndrome is rare, occurring more frequently when high dose gadolinium contrast is administered to patients with severe renal dysfunction [45].

MRI has become the de facto standard for measuring cardiac chamber volumes, in particular those of the left ventricle. CMR is particularly useful in visualizing the right sided heart structures, which are often difficult to image using echocardiography and difficult to image with nuclear medicine studies due to overwhelming signal from the much thicker left ventricle. CMR has been validated in women receiving traztuzumab as part of a chemotherapeutic regimen, against both 2D and 3D echocardiography [31]. Those patients who developed trastuzumab cardiomyopathy further demonstrated late gadolinium enhancement (see below) as well as LV chamber dilation and a reduction in LV EF twelve months into therapy [25]. It remains to be seen if CMR chamber evaluation can predict cardiotoxicity early enough to successfully guide therapy however its accuracy in chamber visualization makes it likely to perform with as much, if not increased, sensitivity as other methods of cardiac chamber imaging such as echocardiography, though with as yet undetermined specificity.

One of the tremendous strengths of MRI is the ability to detect even minor myocardial injury with late gadolinium enhancement. Gadolinium contrast agents are typically not protein bound and diffuse freely into the interstitial space where interaction can occur with interstitial structures. This results in compartmentalization and differential washout kinetics. Gadolinium contrast agent remains in fibrosed, or scarred tissues, for a longer period of time than in healthy tissues. Gadolinium shortens T1 relaxation of surrounding tissue and enables identification and characterization of this tissue utilizing a variety of imaging pulse sequences. Late MR imaging (10-20 minutes following gadolinium administration) utilizing an inversion recovery sequence timed to null healthy myocardium produces bright, easily identifiable areas where myocardial scar is present. This late gadolinium enhancement produces different patterns of enhancement in various disease states that affect the myocardium. For example, cardiac MRI is a powerful imaging modality for the non-invasive diagnosis of myocarditis – an entity in which other imaging modalities often fail [32, 33]. Cardiac MRI, with its unparalleled ability to noninvasively detect fibrosis, may become a boon in the early detection of chemotherapy-induced cardiomyopathies. The utility of late gadolinium enhancement in the context of chemotherapeutic cardiotoxicity remains to be determined. A case series of patients with known trastuzumab associated cardiomyopathy has demonstrated evidence of sub-endocardial late gadolinium enhancement in all patients, with late gadolinium enhancement of the lateral wall in all of these patients [34]. Further, late gadolinium enhancement on cardiac MRI, or lack thereof, may play a role in determining the reversibility of acute cardiotoxicity, and may hint at the mechanism of toxicity in certain agents [35].

## PROGNOSIS

Without exception, prognosis in chemotherapy-associated cardiotoxicity is determined by the presence and degree of cardiac dysfunction at presentation. Efficacy of specific therapy for heart failure associated with chemotherapy is not established, nor is the efficacy of prophylactic regimens. Treatment therefore falls to standard heart failure therapies, including beta adrenergic receptor blockade, modification of the renin-angiotensin-aldosterone pathway with angiotensin converting enzyme inhibitors or angiotensin receptor blocking agents, aldosterone antagonists and diuretics as appropriate [36]. Management of these patients will benefit from involvement of a cardiovascular specialist. 

## RECOMMENDATIONS

Prior to initiation of chemotherapy, baseline cardiovascular health examination should be undertaken, including assessment and management of hypertension, hyperlipidemia, coronary disease and optimization of the patient’s health status for primary prevention of cardiovascular disease. In patients who have known significant cardiovascular disease, such as coronary artery disease associated with left ventricular dysfunction (ischemic cardiomyopathy), valvular heart disease, congenital or other structural heart disease, or arrhythmia, we recommend evaluation by a board certified cardiologist prior to initiation of therapy. When planned chemotherapy is likely to involve an agent associated with significant cardiotoxicity, baseline assessment of left ventricular function and cardiac structure is warranted (See Fig. (**3**)). 

The baseline cardiovascular examination is crucial to risk stratifying and identifying any pre-existing cardiac disease which may influence the therapeutic course, frequency or modality of monitoring through the course of chemotherapy. This initial evaluation may be carried out by an internist or cardiovascular specialist, dependent upon individual level of comfort. If risk factors for cardiovascular disease, particularly coronary artery disease are present, referral to a cardiologist for further evaluation is recommended. If the risk of obstructive coronary disease is high, additional testing may include stress imaging or CT coronary angiography. Once appropriate evaluation and, if necessary, consideration for revascularization and/or medical management of cardiovascular disorders have been implemented, a baseline assessment of cardiac structure and function should be the next step. 

Protocols have been described and utilized in many clinical trials of anti-tumor agents, however these vary significantly in regard to the monitoring methodology, definition of toxicity, and recommendations for dose modification [37-39]. Recommendations for the monitoring of pediatric patients undergoing anthracycline therapy have been published previously, as have guidelines for the monitoring of treated survivors of childhoold malignancy [40, 41]. Data regarding the long term effects of dose or regimen modification based upon such monitoring for cardiotoxicity is lacking; thus the clinical decision making regarding both monitoring strategy and subsequent interventions fall to the treating oncologist, with the option of consultation with a cardiovascular specialist. 

We recommend echocardiography as the imaging modality of choice for initial and follow-up assessment of left ventricular systolic function and structure. As new techniques such as 3D echocardiography and DTI mature, they will likely provide additional diagnostic and prognostic information which is unlikely to be available using other modalities. In patients whom good echocardiographic windows are unavailable, or in whom a question of declining left ventricular function is raised, cardiac MRI would the next recommended option. The reason to move toward cardiac MRI lies in its increased accuracy in determining cardiac chamber volumes, thereby providing more accurate diagnostic information and reducing inter- and intra-observer variability. Perhaps most importantly, the pattern of late gadolinium enhancement frequently yields unique insight into the possible etiology of loss of function. If the patient is not a candidate for MRI, then radionuclide imaging (MUGA) should be considered (see Figure. 4). MRI has the advantage of additional diagnostic benefits as described above with no exposure to ionizing radiation. It is important to note that a typical sestamibi myocardial perfusion scan is a significant exposure to ionizing radiation. Repeated imaging in this manger result in a significant radiation exposure, even if clinically indicated and necessary. No established guidelines describing modality or frequency of evaluation exist, though noninvasive monitoring of cardiac function is endorsed by the AHA/ACC. Once a method for evaluation of LV systolic function is chosen, that method should be utilized henceforth, generally prior to each chemotherapeutic regimen. After the baseline LV function is established, a decision must be made as to the safety of proceeding with the planned chemotherapeutic regimen, and adjustments made as appropriate. This occurs *via* a discussion between the treating oncologist and consulting cardiovascular specialist. 

**Fig. (3):**
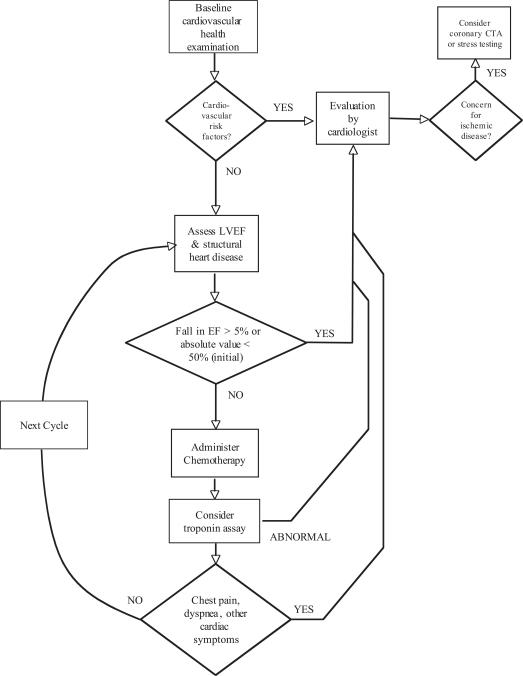
Recommended algorithm for serial monitoring of cardiac function during chemotherapy utilizing cardiovascular imaging techniques. The methodology used to assess LV function is chosen according to the algorithm outlined in Fig. (4). Once a particular modality is chosen, in general, that same method should be used to assess LV function on subsequent cycles.

**Fig. (4):**
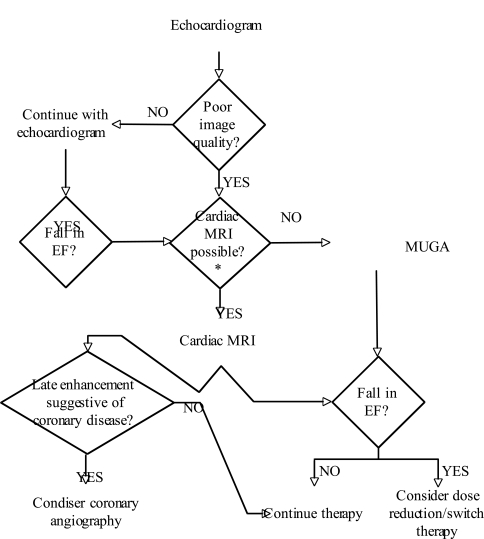
Algortihm for choosing a modality for assessment of left ventricular function. Echocardiography is recommended as the method of choice. If poor image quality limits interpretation, or a fall in EF is detected (see text), then transition to cardiac MRI is recommended. If cardiac MRI is not possible or contraindicated, use of MUGA is indicated. Once a methodology is chosen, we recommend utilizing that methodology for serial assessment. If a fall in EF is detected, transition to the use of cardiac MRI or MUGA to confirm the change is warranted, and if sustained, serial examination with the new modality would be continued. *Aside from availability of appropriate equipment, staff and technicians, contraindications to cardiac MRI include ferromagnetic implants (including certain types of intracranial aneurysm clips), cardiac pacemakers and defibrillators, invasive hemodynamic monitoring devices, certain implanted contraceptive devices, cochlear implants, ferromagnetic shrapnel or other foreign bodies. In the case of gadolinium based contrast administration, contraindications include hepatorenal syndrome, prior liver transplant, and creatinine clearance less than 30 mL/min

The FDA recommends assessment of left-ventricular systolic function prior to each course of anthracycline (specifically in the case of daunorubicin). We would extend this recommendation to all chemotherapeutic agents known to cause long-term cardiac dysfunction. No concrete guideline has been published for many of the chemotherapeutic agents regarding a quantum of change in LV systolic function. For example, a reduction in LVEF to an absolute level <55% or a 10% decline in LVEF indicates deterioration of cardiac function as defined by the CREC [3]. It is recommended that all anthracyclines be discontinued below an absolute fractional shortening of 26% [42]. Assessment of LV function should occur, ideally by the same modality as originally chosen, prior to each cycle of chemotherapy. We recommend reassessment by a cardiovascular specialist for a fall in EF of greater than 5%, or to an absolute value less than 55%. A decline in LVEF beyond the criteria listed above would require re-assessment by the cardiovascular specialist, and re-evaluation of the therapeutic regimen. Figure 3, above describes the use of MRI as a follow-up to a fall in LVEF identified by either echocardiography or MUGA. Cardiac MRI can provide more accurate chamber quantification and may detect or rebuke subtle changes in ventricular function with higher accuracy. Once a fall in LVEF has been confirmed with cardiac MRI, we recommend continuing further assessment with MRI prior to each cycle of chemotherapy moving forward. 

When high dose chemotherapy is administered, we recommend evaluation with serial serum troponins immediately following and at 12, 24, 36 and 72h after the end of a each cycle of chemotherapy when feasible (such as in hospitalized patients). A positive troponin assay (troponin I > 0.08 ng/dL) should trigger a referral to a cardiovascular specialist, as this may serve as a marker for future cardiac dysfunction and consideration should be given to dose reduction, change in regimen, or addition of cardioprotective agents. Follow up one month later with a second troponin assay is also warranted in those patients with early elevations in serum troponin, to further risk stratify those patients at risk for more severe long term LV dysfunction. 

Of course, after each dose of chemotherapy a clinical assessment is paramount to detect any clinical signs or symptoms resulting from underlying cardiovascular dysfunction. Presence of chest pain not easily attributable to a non-cardiac cause, dyspnea, edema, or other evidence of ischemia or congestive heart failure would trigger immediate re-evaluation by the cardiologist. 

## CONCLUSION

As patient survival continues to improve in treatment of malignancy, long term toxicity becomes a larger issue. With accumulating evidence behind successful therapy of some cardiac toxicities following chemotherapy, early identification takes on increasing importance. Presently, no evidenced based guidelines exist to guide monitoring for cardiovascular toxicity both during and following the administration of chemotherapeutic agents. It is further important to avoid toxicity secondary to a monitoring strategy, which could itself have long term consequences, either in the form of organ dysfunction or induction of secondary malignancies. In this review we have presented several techniques for detection and monitoring for cardiotoxicity during and following chemotherapuetic administration, and suggest an algorithm to assist the clinician in early detection of cardiac toxicity.

## Figures and Tables

**Fig. (2):**
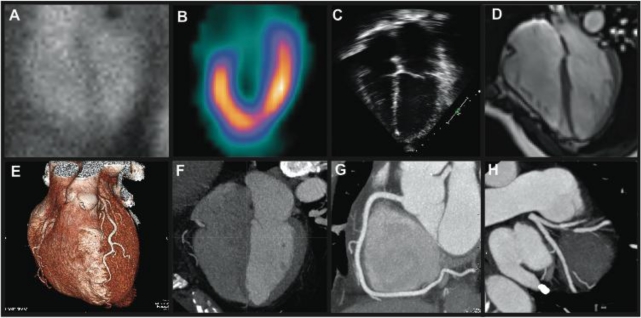
Noninvasive assessment of cardiac structure and function. Multiple-gated acquisition (MUGA) images of the right and left ventricles (**A**). Single photon emission computed tomography (SPECT) assessment of the left ventricle (**B**). Echocardiography 4-chamber view of the right and left ventricles as obtained from an apical window (**C**). Cardiac magnetic resonance (CMR) images of the right and left ventricles obtaining utilizing a 1.5 Tesla MRI scanner (**D**). Coronary CT angiography images, including a volume-rendered image (**E**), multiplanar reconstruction of the right and left ventricles (**F**), and maximum intensity projections of the right coronary artery (**G**) and left anterior descending coronary artery (**H**).
